# Uncovering the Translational Regulatory Activity of the Tumor Suppressor BRCA1

**DOI:** 10.3390/cells9040941

**Published:** 2020-04-10

**Authors:** Elise Berthel, Anne Vincent, Lauriane Eberst, Adrian Gabriel Torres, Estelle Dacheux, Catherine Rey, Virginie Marcel, Hermes Paraqindes, Joël Lachuer, Frédéric Catez, Lluis Ribas de Pouplana, Isabelle Treilleux, Jean-Jacques Diaz, Nicole Dalla Venezia

**Affiliations:** 1 Inserm U1052, CNRS UMR5286, Centre de Recherche en Cancérologie de Lyon, Université de Lyon 1, Centre Léon Bérard, F-69008 Lyon, France; elise.berthel@inserm.fr (E.B.) ; anne.vincent@lyon.unicancer.fr (A.V.) ; estelle.dacheux@pasteur.fr (E.D.) ; Virginie.MARCEL@lyon.unicancer.fr (V.M.) ; Hermes.PARAQINDES@lyon.unicancer.fr (H.P.) ; frederic.catez@lyon.unicancer.fr (F.C.) ; jeanjacques.diaz@lyon.unicancer.fr (J.-J.D.); 2 Centre Léon Bérard, Medical Oncology Department, Université de Lyon 1, F-69008 Lyon, France; lauriane.eberst@lyon.unicancer.fr; 3 Institute for Research in Biomedicine (IRB Barcelona), The Barcelona Institute of Science and Technology, Baldiri Reixac, 10, 08028 Barcelona, Spain; adriangabriel.torres@irbbarcelona.org (A.G.T.); lluis.ribas@irbbarcelona.org (L.R.d.P.); 4 ProfileXpert, UNIV-US7 INSERM-UMS 3453 CNRS, F-69000 Lyon, France; catherine.rey@inserm.fr (C.R.); joel.lachuer@univ-lyon1.fr (J.L.); 5 Catalan Institution for Research and Advanced Studies (ICREA), Passeig Lluis Companys 23, 08010 Barcelona, Spain; 6 Department of Translational Research and Innovation, Centre Léon Bérard, F-69008 Lyon, France; isabelle.treilleux@lyon.unicancer.fr

**Keywords:** BRCA1, translation, breast cancer, tumor suppressor

## Abstract

BRCA1 inactivation is a hallmark of familial breast cancer, often associated with aggressive triple negative breast cancers. BRCA1 is a tumor suppressor with known functions in DNA repair, transcription regulation, cell cycle control, and apoptosis. In the present study, we demonstrate that BRCA1 is also a translational regulator. We previously showed that BRCA1 was implicated in translation regulation. Here, we asked whether translational control could be a novel function of BRCA1 that contributes to its tumor suppressive activity. A combination of RNA-binding protein immunoprecipitation, microarray analysis, and polysome profiling, was used to identify the mRNAs that were specifically deregulated under BRCA1 deficiency. Western blot analysis allowed us to confirm at the protein level the deregulated translation of a subset of mRNAs. A unique and dedicated cohort of patients with documented germ-line BRCA1 pathogenic variant statues was set up, and tissue microarrays with the biopsies of these patients were constructed and analyzed by immunohistochemistry for their content in each candidate protein. Here, we show that BRCA1 translationally regulates a subset of mRNAs with which it associates. These mRNAs code for proteins involved in major programs in cancer. Accordingly, the level of these key proteins is correlated with BRCA1 status in breast cancer cell lines and in patient breast tumors. ADAT2, one of these key proteins, is proposed as a predictive biomarker of efficacy of treatments recently recommended to patients with BRCA1 deficiency. This study proposes that translational control may represent a novel molecular mechanism with potential clinical impact through which BRCA1 is a tumor suppressor.

## 1. Introduction

Breast cancer is the most common cancer worldwide, accounting for 2.1 million new cases and 627,000 deaths worldwide in 2018 [1]. Approximately 5% of these patients carry a germline pathogenic variant in BRCA [2,3,4] with a risk of developing breast cancer reaching 72% by age of 80 years [5,6]. Additionally, in a significant proportion of sporadic breast tumors, Breast Cancer 1 (BRCA1) promoter hypermethylation, transcription repression, or somatic BRCA1 pathogenic variants are responsible for its inactivation [7,8]. BRCA1 pathogenic variant carriers are more likely to be diagnosed with a triple negative breast cancer (i.e., estrogen-receptor negative, progesterone-receptor negative, and human epidermal growth factor receptor 2 (HER2) negative), which is associated with poor prognosis due to limited therapeutic options [9]. The identification of BRCA1 pathogenic variant carriers is of the most importance since these patients can now benefit from preventive treatments (early detection of contra-lateral breast cancer, prophylactic mastectomy, or oophorectomy) and most importantly from innovative treatments, including poly (adenosine diphosphate-ribose) polymerase (PARP) inhibitors like olaparib [10].

The BRCA1 protein is a tumor suppressor. It maintains genomic integrity through multiple functions; the best documented being those related to DNA repair [11,12,13,14]. However, other functions participate in its tumor suppressive capacity including chromatin remodeling, cell cycle regulation, transcription regulation, mRNA splicing, and apoptosis [11,15,16]. Furthermore, we previously showed that BRCA1 was implicated in translational regulation [17,18]. Whether translational control could be a novel function of BRCA1 contributing to its tumor suppressor capabilities remains however to be assessed.

Translational control is a master regulator of gene expression and its deregulation is a hallmark of cancer. Here, we report translational regulation as a novel pivotal function of BRCA1 that may represent a novel mechanism through which BRCA1 exerts its tumor suppressive activity. BRCA1 protein controls the translation of mRNAs with which it interacts. Upon BRCA1 inactivation, Adenosine Deaminase, tRNA Specific 2 (ADAT2), Cyclin L2 (CCNL2), Protein DBF4 homolog B (DBF4B), and Tripartite Motif Containing 45 (TRIM45) mRNAs display altered translation efficiencies with concomitant changes of their protein levels. In mammary tumors from patients mutated for BRCA1, expression of these proteins is also impacted as a consequence of BRCA1 loss of function. Our study uncovers translational control as a novel BRCA1 function with potential clinical impact.

## 2. Materials and Methods

### 2.1. Chemicals

Refer to Supplementary Material and Methods for details.

### 2.2. Case Selection

Patients with clinical history suggestive of BRCA1 or BRCA2 pathogenic variants (i.e., predictive risk of pathogenic variant according to the Eisinger score superior to 3 [19] were referred to a geneticist, in order to detect a germline BRCA1 or BRCA2 pathogenic variant. Case selection is detailed in Supplementary Material and Methods. 

### 2.3. Tissue Microarray (TMA) and Immunohistochemistry (IHC)

Breast tumors from each of the 3 groups (exploratory mutant (MT) BRCA1, exploratory wild-type (WT) BRCA1, and validation MT BRCA1) were inserted into 3 different tissue microarray (TMA) blocks, each tumor specimen being divided and inserted in triplicate. Four microns thick unstained sections were cut from each TMA block and mounted on slides. The slides were processed for immunohistochemistry (IHC) and immunostained as described in Supplementary Material and Methods.

The proportion of positively stained tumor cells in each section was graded as follow: 0, ≤ 10%; 1, 20–40%; 2, 50–80%; and 3, > 80% positive cells. The staining intensity was recorded on a scale comprised between 0 and 3, corresponding to null to high intensity of the signal. The scores were calculated as follows: staining intensity × proportion of positively stained cells. A Chi-square statistical analysis was conducted to test for the significant difference between cohort scores.

### 2.4. Cell Culture

Refer to Supplementary Material and Methods for details.

### 2.5. Transfection with Plasmids

For transfection, MCF-7 and MDA-MB-231 cells were plated at 2 × 10^6^ cells per 10 cm diameter dish 24 h before transfection with 4 µg of plasmid and 20 µL of ExGen 500 (Euromedex, Souffelweyersheim, France). Twenty four hours after transfection, cells were harvested. The pCDNA3 plasmid expressing full length BRCA1 protein (BRCA1) was previously described [20].

### 2.6. RNA Interference (RNAi)

The siRNA duplexes were purchased from Eurofins Genomics (Ebersberg, Germany) and provided as purified and annealed duplexes. The sequence of the siRNA against BRCA1 was si-BRCA1 5′-GGAACCUGUCUCCACAAAG-3′ [17,20]. The siRNA used as control was designed against luciferase: si-Ctrl 5′- CGUACGCGGAAUACUUCGA-3′.

MCF-7, MDA-MB-231, and HMECs were plated at 2 × 10^6^ cells per 10 cm diameter dish 24 h before transfection. Cells were transfected with 200 pmol/dish of siRNA and 16 µL/dish of Lipofectamine RNAiMAX (Thermo Fisher Scientific, Bourgoin-Jallieu, France) using the protocol provided by the supplier. Cells were harvested 72 h after transfection.

### 2.7. Immunoblotting

Immunoblotting was performed as previously reported [18]. Refer to Supplementary Material and Methods for details.

### 2.8. Isolation of Polysomes and Total Cytoplasmic RNA

Isolation of polysomes and total cytoplasmic RNA was carried out as previously [18]. Refer to Supplementary Material and Methods for details.

### 2.9. RNA-Binding Protein Immunoprecipitation (RIP)

RNA-binding protein immunoprecipitation (RIP) experiments were performed mainly using the standard reference protocols including the relevant non-specific control [21]. Refer to Supplementary Material and Methods for details.

### 2.10. Microarray Analysis

RNA isolation following immunoprecipitation with anti-BRCA1 antibody and control antibody (NR) was performed in triplicate as described in the “RNA-binding protein immunoprecipitation (RIP)” section.

Microarray processing and data analysis was performed at the ProfileXpert core facility (Lyon, France). Details are provided in Supplementary Material and Method.

The complete set of raw and normalized data is available at the GEO database under accession number GSE119886 (https://www.ncbi.nlm.nih.gov/geo/query/acc.cgi?acc=GSE119886).

For each replicate, the probe intensities issued from the BRCA1 and NR immunoprecipitations were obtained. The obtained data were normalized with Affymetrix Expression Console software using the RMA statistical algorithm. Control expression values (NR) were subtracted from their respective sample counterparts on a probe set basis; the 3 replicates were then averaged. Only genes showing an immunoprecipitation (IP) ratio IP BRCA1/IP NR (Fc) greater than 1.5 and a *p*-value lower than 0.05 were retained.

The retained genes of interest were listed and classified according to their biological process (BP), cellular compartment (CC), and molecular function (MF) using DAVID (Database for Annotation, Visualization and Integrated Discovery) on 10th of March 2017 [22] (https://david.ncifcrf.gov/summary.jsp).

### 2.11. Quantitative RT-PCR

#### 2.11.1. RIP Analysis

Three additional replicates of RIP, including inputs, were dedicated to RT-qPCR. RNA extracted from RIP (IP BRCA1 and IP NR) and RNA extracted from the corresponding inputs, was reverse-transcribed and pre-amplified with the Nugen Ovation WTA System (TECAN, Redwood City, USA). Quantitative real-time PCR (qPCR) was carried out using the light cycler 480 II real-time PCR thermocycler (Roche, Meylan, France). The experimental protocol consisted in an initial polymerase activation for 10 min at 95 °C followed by an amplification program of 10 sec at 95 °C, 10 sec at 60 °C, and 10 sec at 72 °C, for 45 cycles. Expression of mRNA was quantified using the LightCycler 480 SYBR Green I Master Mix (Roche) and normalized using POP4 and Actin expression according to the 2-ΔΔCt method. Immunoprecipitated RNA was expressed as fold enrichment over input compared to enrichment with non-relevant antibody (NR) used as a control; fold enrichment Fe = (IP BRCA1 − IP NR)/Input.

#### 2.11.2. Polysome Profile Analysis

Total and polysomal RNA prepared as described above, from BRCA1-depleted and control cells, were purified and submitted to DNase digestion using NucleoSpin RNA XS Clean-up columns (Macherey-Nagel, Hoerdt, France) following the manufacturer’s instructions. Next, 250 ng of total RNA were reverse transcribed using the M-MLV RT kit and random primers (Thermo Fisher Scientific, Bourgoin-Jallieu, France), according to the manufacturer’s instructions. For each RNA type (polysome RNA or total RNA prepared from the same cytoplasmic extract as described above), a ratio between si-BRCA1 and si-Ctrl sample was calculated. The ratios for polysomal RNA (polyRNA = si-BRCA1/si-Ctrl) and for total RNA (totRNA = si-BRCA1/si-Ctrl) were calculated. The polyRNA/totRNA ratio was then calculated and defined as translational efficiency (Te). Only genes showing a Te greater than 1.4 or lower than −1.4 in the three replicates were retained. 

Primers were designed using the Primer-Blast software (National Centre for Biotechnology Information/NCBI, Bethesda, USA; http://www.ncbi.nlm.nih.gov/tools/primer-blast) and purchased from Eurofins Genomics (Ebersberg, Germany). For each gene, primers were designed within exons that displayed high exon probe intensities on arrays. Genes tested for polysomal profiling and RIP, and primers, are listed in the Table S1.

### 2.12. Quantification of A-to-I Editing

Quantification of tRNA editing was determined as previously described [23] with minor modifications. Refer to Supplementary Material and Methods for details. The RNAseq data generated for this study has been deposited at the EBI European Nucleotide Archive (Accession number PRJEB28622).

### 2.13. Statistical Analysis

Refer to Supplementary Material and Methods for details.

## 3. Results

### 3.1. BRCA1 Associates with mRNAs

**Figure 1:**
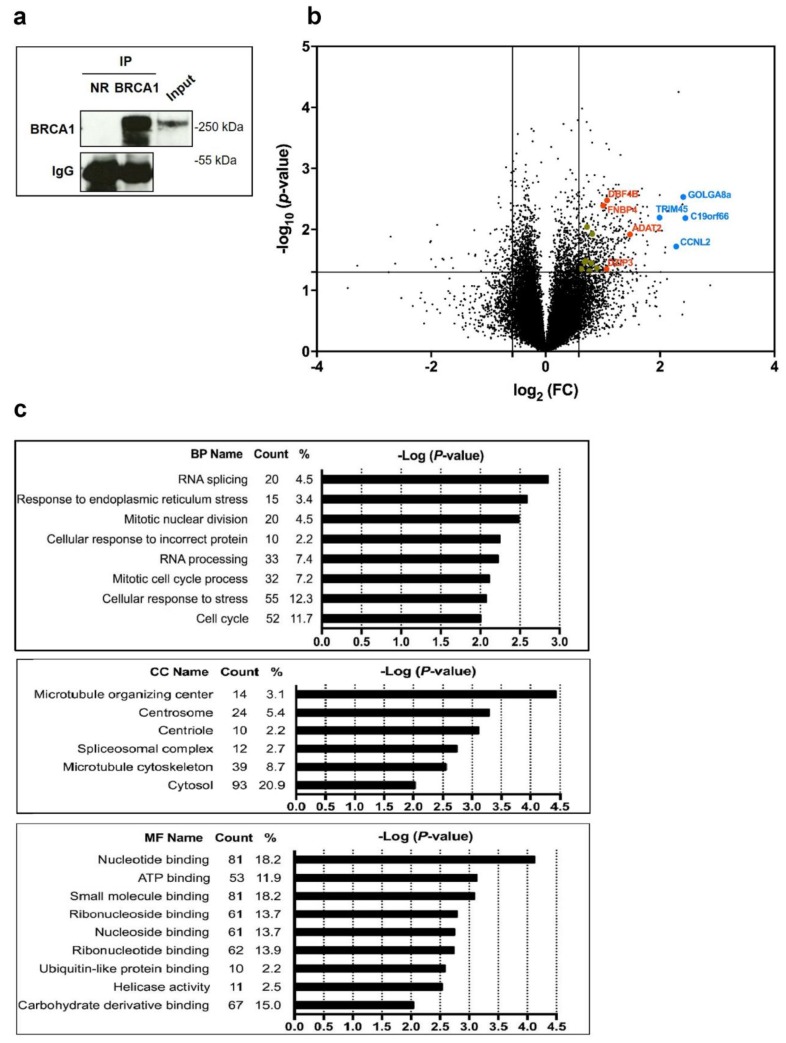
Identification of BRCA1-associated mRNAs using large-scale analysis. RNA-binding protein immunoprecipitation (RIP) performed in triplicate followed by microarray analysis. (**a**) Immunoblots showing that BRCA1 is immunoprecipitated in MCF-7 cells. Immunoprecipitation with anti-BRCA1 antibody (BRCA1) and with a control IgG (NR). (**b**) Microarray analysis of the BRCA1-associated mRNA isolated by RIP. Lines indicate –log10 (*p*-value) ≥ 1.3 and abs (log2 (Fc)) ≥ 0.6 for *p* ≤ 0.05 and abs (Fc) ≥ 1.5 respectively. Among the mRNAs displaying a fold change (Fc) above 1.5, the 16 mRNA of interest (listed in Figure 2a) were colored in gray (1.5 ≤ Fc < 2.0), orange (2.0 ≤ Fc < 3.0), and blue (3.0 ≤ Fc ≤ 5.4). The name of the 8 genes with the most changes (colored in blue and orange) was included. (**c**) Gene ontology analysis of mRNA bound to BRCA1, using DAVID (Database for Annotation, Visualization and Integrated Discovery). BP: biological process; CC: cellular compartment; MF: molecular function.

Having previously shown that BRCA1 depletion affects protein synthesis and translational efficiency [17,18], we here investigated which mRNA were associated with BRCA1 protein. We used an RNA binding protein immunoprecipitation (RIP) assay followed by microarray analysis of the BRCA1-bound mRNA. Three total lysates of MCF-7 cells were independently treated. Each lysate was split in two equal portions. One was incubated with anti-BRCA1 antibody (BRCA1) while the second was incubated with control antibody (NR). We first confirmed efficient immunoprecipitation of BRCA1 by Western blot analysis (Figure 1a).

The microarray analysis was then performed for each replicate, and the probe intensities issued from the BRCA1 and NR immunoprecipitations were averaged. Thus, for each mRNA, the binding to control IgG and the binding to anti-BRCA1 antibody were measured in parallel. Only mRNAs showing an IP BRCA1/IP NR ratio (Fc) greater than 1.5 and a *p*-value lower than 0.05 were retained. Overall, among the 29591 mRNA detected on chip, 498 transcripts representing 2% of mRNAs expressed in MCF7 cells, were 1.5 fold more abundant in BRCA1 immunoprecipitates compared to control immunoprecipitates (Figure 1b). To provide a first view of the biological properties of the proteins encoded by these mRNAs, we conducted a gene ontology analysis. The identification of the most enriched biological processes, molecular functions, and cellular compartments associated with these mRNAs, revealed that most of the enriched processes have previously been related to BRCA1 tumor suppressor activity such as stress response, RNA splicing, and cell cycle regulation. In addition, we noticed that the enriched cellular compartments and molecular function, namely cytosol and nucleotide binding, have been poorly associated with BRCA1 activity up to now (Figure 1c).

**Figure 2:**
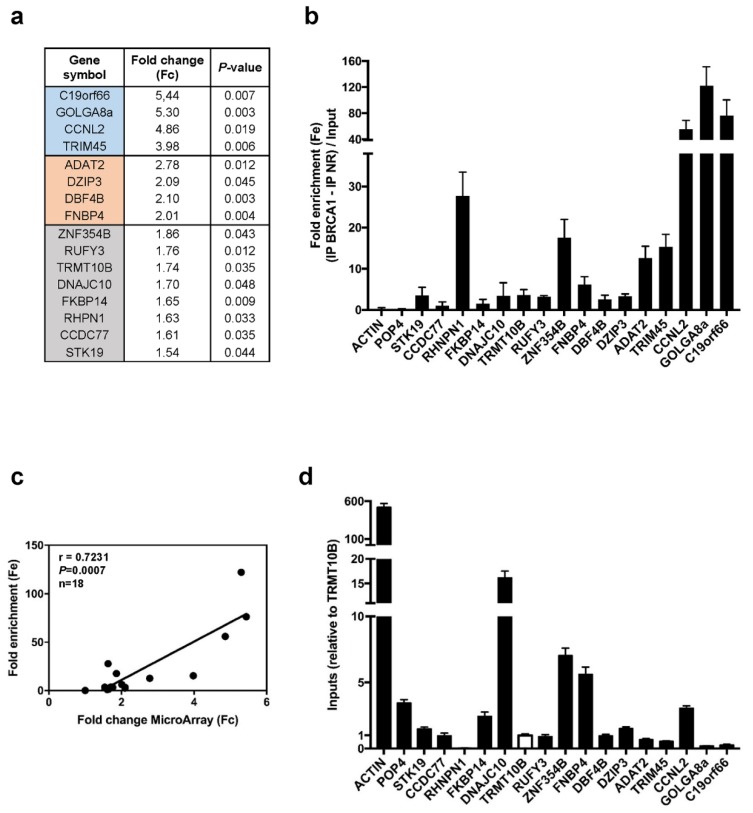
Analysis of a representative subset of BRCA1-associated mRNAs. RIP of three new independent replicates followed by RT-qPCR. (**a**) Subset of 16 BRCA1-associated mRNAs spanning the full range of Fc observed in Figure 1b. Colors indicate the range of Fc obtained by microarray. (**b**) Fold enrichment (Fe) of the 16 BRCA1-associated mRNAs determined by RT-qPCR. Data are expressed as means ± SEM. *n* = 3. (**c**) Correlation between Fc calculated from the Affymetrix array and Fe measured by RT-qPCR. Mean RIP fold change obtained by microarray (Fc) and mean RIP fold enrichment obtained by RT-qPCR (Fe) were plotted and their correlation was assessed using the Spearman test. A significant correlation was observed. (**d**) Quantification by RT-qPCR of each BRCA1-associated mRNA in total extracts of MCF-7 cells (inputs). The TRMT10B value was arbitrarily set at 1 (white bar). Data are expressed as means ± SEM. *n* = 3.

Next, we ascertained that the Fc observed for these mRNAs was correlated with their binding to BRCA1 and not with their cytoplasmic abundance. We selected a subset of 16 mRNAs spanning the full range of the Fc (Figure 2a).

We conducted three new independent RIP assays to measure the fold enrichment (Fe) of each mRNA normalized against its total abundance (input) in the whole cell (Figure 2b). A significant and positive correlation was observed between Fc obtained by microarray analyses and Fe (Figure 2c). The absence of correlation between RIP Fe and mRNA levels (Figure 2d) underlines that BRCA1 associates with these mRNA independently of their cytoplasmic quantity.

### 3.2. BRCA1 Controls Translation of a Subset of BRCA1-Associated mRNAs

**Figure 3:**
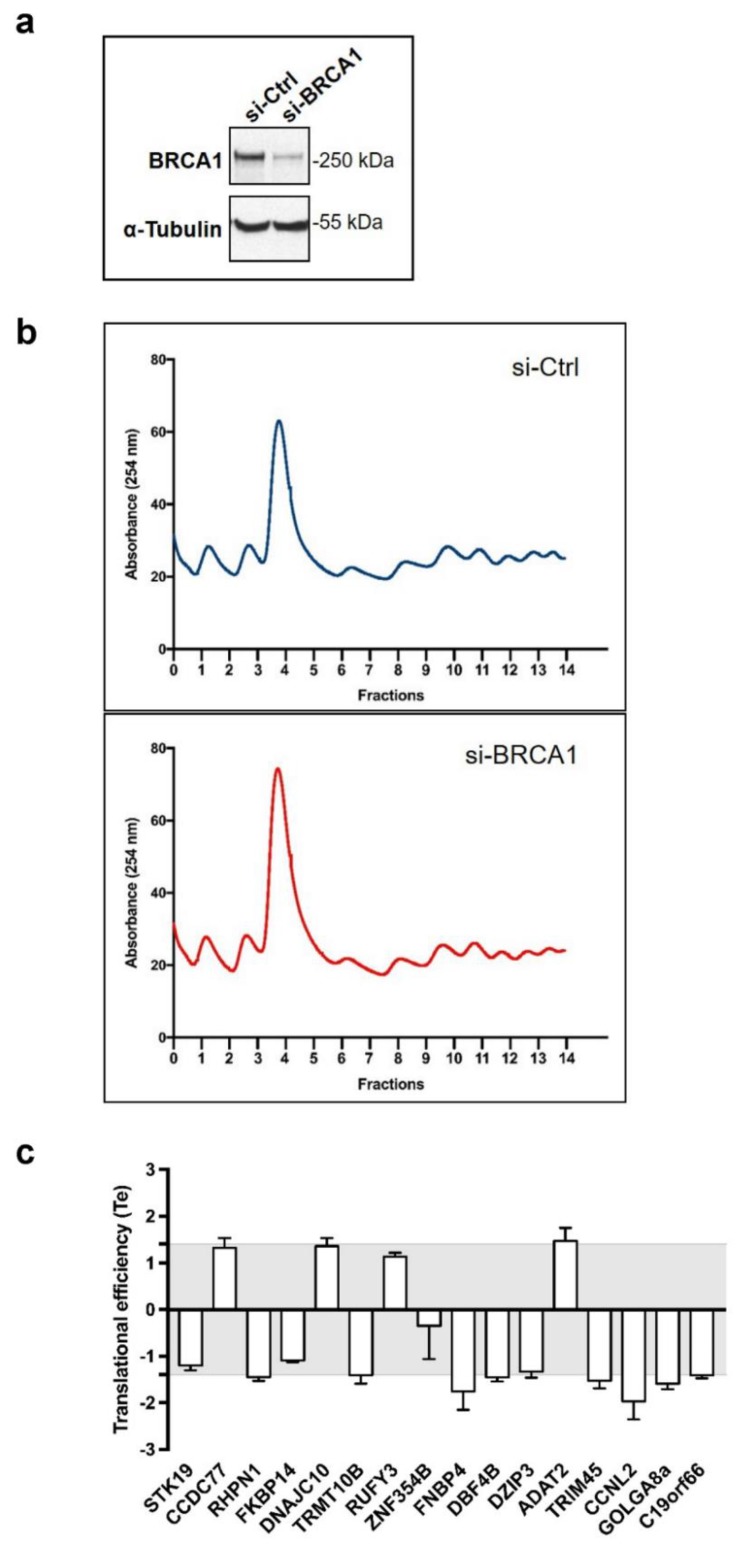
BRCA1 controls translation of a subset of BRCA1-associated mRNAs. (**a**) Immunoblots confirming siRNA inhibition of BRCA1 (si-BRCA1) when compared with control siRNA (si-Ctrl) in MCF-7 cells. α-Tubulin served as a loading control. (**b**) Polysomal profiles of MCF-7 cells in response to depletion of BRCA1. 40S and 60S ribosomal subunits, 80S ribosomes, and polysomes were separated by ultracentrifugation on sucrose gradients. One representative polysome profile of cells transfected with control siRNA (si-Ctrl) and with BRCA1-targeting siRNA (si-BRCA1) is shown. (**c**) Analysis of the translational efficiency (Te) of the 16 BRCA1-associated mRNAs identified in Figure 2. For BRCA1-depleted cells (si-BRCA1) and for control cells (si-Ctrl), fractions 7–14 containing polysomal material were recovered, pooled, and processed for RNA extraction. In parallel, a portion of the cytoplasmic extract was kept unprocessed to perform total RNA extraction from si-BRCA1 and from si-Ctrl cells. For each of the 16 mRNA of interest, RT-qPCR was performed on total RNA and polysomal RNA from si-BRCA1 and si-Ctrl cells. The Te was determined by calculating the following ratio: (change in abundance in polysomal mRNA in the absence and presence of BRCA1)/(change in abundance in total mRNA in absence and presence of BRCA1). Data are expressed as means ± SEM. *n* = 3.

To investigate whether BRCA1 regulates the translation of mRNA with which it associates, we silenced BRCA1 expression in MCF-7 cells using a previously described BRCA1-targeting siRNA [17,20], which achieved clear BRCA1 depletion (Figure 3a).

We assessed the translational efficiency (Te) of each of the 16 mRNAs by analyzing mRNA abundance in polysomal fractions (i.e., actively translated mRNA) related to the total cytoplasmic mRNA, and compared control and BRCA1-depleted cells, in three independent experiments (Figure 3b).

First we performed a polysome profiling following three sequential steps as follows. (1) A large fraction (80%) of the cytoplasmic extract was loaded on top of a 10%–40% sucrose gradient. After ultracentrifugation, untranslated mRNAs were present in the top fractions whereas 40S and 60S free subunits, 80S ribosomes, and polysome-associated mRNAs were distributed along the bottom part of the gradient. (2) The gradient was then collected using a flow cell coupled to a spectrophotometer to measure and register continuously the concentration of RNA in each fraction of the gradient represented by the OD at 254 nm. The gradient was collected into 14 fractions of equal volumes. The variations of RNA concentration were reported as a function of the gradient fractions and thus determine which fractions contain free ribosomal subunits, monosomal, or polysomal material. (3) Fractions containing polysomal material were recovered, pooled, and processed for RNA extraction. In parallel, 20% of the cytoplasmic extract was directly processed for RNA extraction.

By comparing profiles obtained with BRCA1-depleted cells with those of control cells, we observed that they were similar, notably regarding the fractions 7–14 containing actively translated mRNA, thus indicating that translation was not grossly altered by BRCA1.

Next, for each of the 16 mRNA of interest (see Figure 2), RT-qPCR were performed on total RNA from BRCA1-depleted and control cells and on polysomal RNA from BRCA1-depleted and control cells. Then the Te was calculated following a three steps procedure. (1) We first investigated the variations in mRNA content within the total cytoplasmic RNA fraction in the presence and absence of BRCA1, to determine whether BRCA1 modulates the amounts of cytoplasmic mRNAs reflecting their rates of synthesis, transport, and stability (totRNA = si-BRCA1/si-Ctrl). (2) Then, to evaluate how BRCA1 affects mRNA recruitment within polysomes, we analyzed the amount of polysomal RNA in the presence and absence of BRCA1 (polyRNA = si-BRCA1/si-Ctrl). (3) Finally, we determined the Te of each mRNA by calculating the following ratio: (change in abundance in polysomal mRNA)/(change in abundance in total mRNA) (Te = polyRNA/totRNA). Therefore, for each mRNA expressed, the Te value reveals the change in its association with polysomes independently to any change in its total cytoplasmic amount.

By applying cut-off values of 1.4, we found that loss of BRCA1 deregulated the translation of 7 out of the 16 mRNAs analyzed (Figure 3c) by impacting their association with polysomes independently of their abundance in the total cytoplasmic fraction (Figure S1). This cut-off value of 1.4 allowed selecting a number of mRNA, which was found to (i) further analyze by Western blot and (ii) more importantly further the investigation of their biological relevance using IHC on human breast cancer samples.

Taken together, these results established a set of seven mRNAs that were translationally controlled by the BRCA1 protein, with ADAT2 being upregulated, and CCNL2, DBF4B, FNBP4, GOLGA8a, RHPN1, and TRIM45 downregulated upon BRCA1 depletion.

### 3.3. BRCA1 Controls ADAT2, CCNL2, DBF4B, and TRIM45 Proteins in Mammary Epithelial Cell Lines

**Figure 4:**
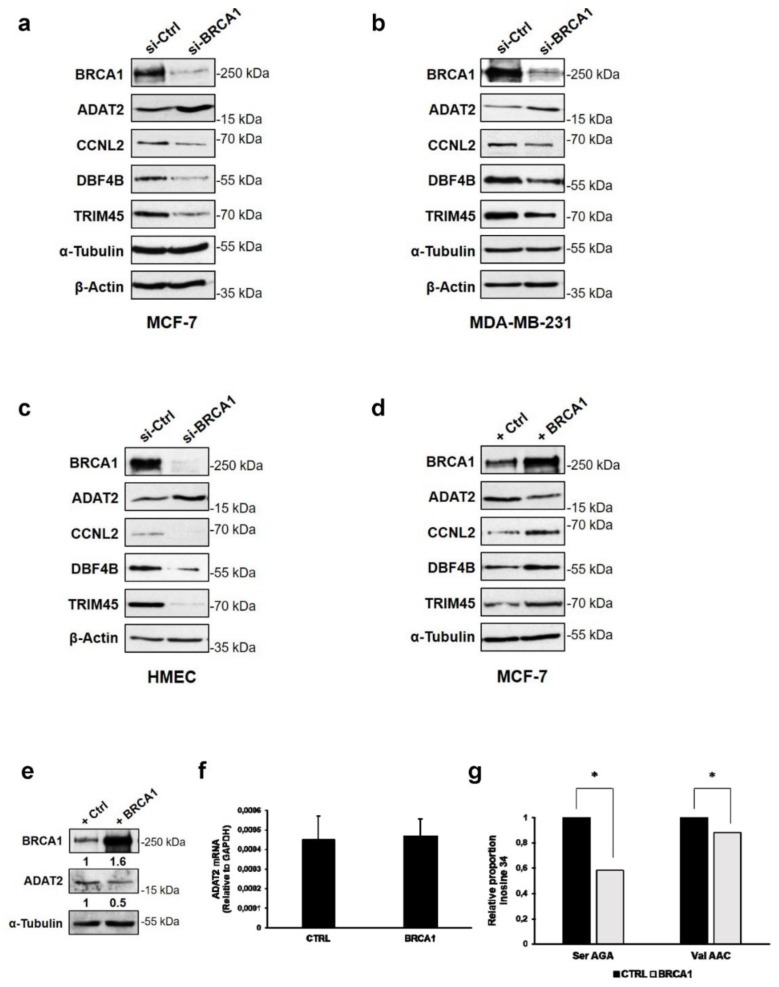
BRCA1 controls ADAT2, CCNL2, DBF4B, and TRIM45 proteins in mammary epithelial cell lines. (**a**–**c**) Immunoblot analyses of ADAT2, CCNL2, DBF4B, and TRIM45 proteins from MCF-7 cells (**a**), MDA-MB-231 cells (**b**), and HMECs (**c**) transfected with BRCA1-targeting siRNA (si-BRCA1) or control siRNA (si-Ctrl). (**d**) Immunoblot analysis of ADAT2, DBF4B, CCNL2, and TRIM45 proteins from MCF-7 cells transfected with BRCA1-expressing plasmid (+BRCA1) or empty plasmid as control (+Ctrl). β-actin and α-tubulin were used as loading controls. (**e**) Immunoblot showing increased levels of BRCA1 and decreased levels of ADAT2 in MCF-7 cells transfected with BRCA1-expressing plasmid (+BRCA1) compared with control (+Ctrl). Numbers indicate the quantity of BRCA1 and ADAT2 in BRCA1-enriched cells compared to control cells and normalized against α-Tubulin. (**f**) RT-qPCR analysis of ADAT2 mRNA in MCF-7 cells overexpressing BRCA1 (BRCA1) or a control vector (CTRL). Experiments were performed as previously described [23,24]. *n* = 2. (**g**) Relative proportion of inosine found at position 34 in tRNASerAGA and tRNAValAAC in BRCA1-enriched (BRCA1) compared to control (CTRL) MCF-7 cells. Data are expressed as means ± SEM. *n* = 2. Results of the Fisher’s Exact Test are indicated by * *p* < 0.05.

Next, we investigated whether modifications in translational efficiency of these mRNA upon BRCA1 depletion lead to changes in the corresponding protein levels. Due to antibody limitations, we could only perform immunoblots of four proteins, namely ADAT2, CCNL2, DBF4B, and TRIM45. We found that BRCA1 depletion induced an increase in the ADAT2 protein and conversely reduced CCNL2, DBF4B, and TRIM45 levels in MCF-7 cells, changes that correlated with the translational efficiency trend (Figure 4a).

These findings were corroborated in other tumoral (MDA-MB-231) and non-tumoral (HMECs) mammary cells (Figure 4b,c), indicating that BRCA1 deficiency alters the translation of selected mRNAs and further affects the quantity of their protein products. Conversely, overexpression of BRCA1 in MCF-7 cells decreased ADAT2 and increased CCNL2, DBF4B, and TRIM45 protein levels (Figure 4d).

To further ascertain the impact of BRCA1 overexpression on the function of the ADAT2 protein in MCF-7 cells, we examined changes in anticodon inosine abundance. Indeed, since ADAT2 generates inosine from adenosine at position 34 in the anticodon of tRNA [23], we quantified tRNA A34-to-I34 editing by comparing I34 levels in control MCF-7 cells and in those overexpressing BRCA1. We found that overexpression of BRCA1 (Figure 4e), while not affecting ADAT2 mRNA levels (Figure 4f), but reducing ADAT2 protein levels (Figure 4e), significantly reduced the levels of I34 in at least two substrates of ADAT2: tRNASerAGA and tRNAValAAC (Figure 4g). Importantly, this reduction is equivalent to that reported in HEK293T cells upon ADAT2 depletion [23]. These results show that BRCA1 controlled the ADAT2 function by translationally modulating ADAT2 abundance.

### 3.4. Altered Expression of ADAT2, CCNL2, DBF4B, and TRIM45 Proteins in BRCA1 Deficient Human Breast Cancers

Given the functional implications of BRCA1 inactivation on the translation of ADAT2, CCNL2, DBF4B, and TRIM45, we wondered whether the levels of these proteins were correlated to BRCA1 status in breast tumors. We set up cohorts of patients with invasive breast cancers and documented germ-line BRCA1 and BRCA2 pathogenic variant statuses (Table 1). 

We analyzed by immunohistochemistry (IHC) the expression of ADAT2, CCNL2, DBF4B, and TRIM45 in an exploratory cohort of 45 tumor samples, 26 from patients with no BRCA1 pathogenic variant (WT BRCA1), and 19 from patients with documented BRCA1 pathogenic variant (MT BRCA1) hampering BRCA1 protein production. Since patients in the MT BRCA1 and WT BRCA1 groups were matched according to other histoprognostic factors (Scarff Bloom and Richardson (SBR) grade, hormonal status, and histological subtype), BRCA1 expression was the only discriminant factor between the two groups, enabling the comparison of ADAT2, CCNL2, DBF4B, and TRIM45 expressions (Table 1).

**Figure 5:**
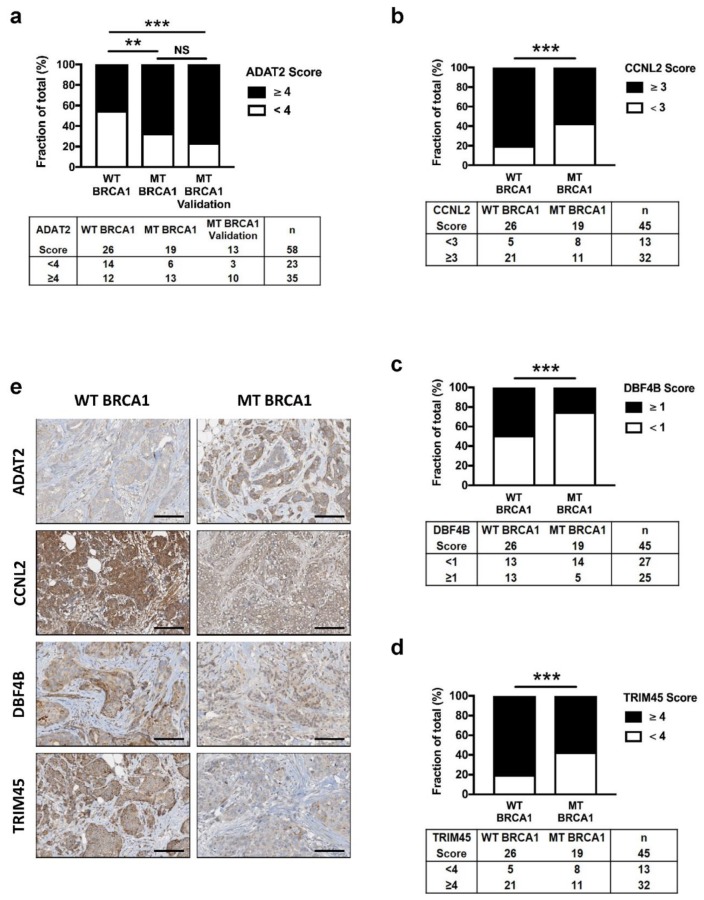
Altered expression of ADAT2, CCNL2, DBF4B, and TRIM45 proteins in BRCA1 deficient human breast cancers. (**a**–**d**) Score of ADAT2 (**a**), CCNL2 (**b**), DBF4B (**c**), and TRIM45 (**d**) in breast cancer samples of 45 patients (exploratory cohort): 26 patients non-mutated (WT BRCA1) and 19 patients mutated (MT BRCA1) for BRCA1. For ADAT2, a validation cohort of 13 BRCA1 mutated patients (MT BRCA1 validation) was additionally analyzed. Stacked bars represent the fraction of total tumor samples (expressed in %) from a tissue microarray (TMA) with low (white bar) and high (black bar) scores. Results of Chi-square test are indicated by NS = non-significant; ** *p* < 0.01; *** *p* < 0.001. Corresponding number of samples are presented under histograms. (**e**) Representative TMA immunohistochemical images demonstrating differential ADAT2, CCNL2, DBF4B, and TRIM45 staining between BRCA1 mutated (MT BRCA1) and BRCA1 non-mutated (WT BRCA1) tumors. Scale bars = 100 µm.

Staining scores showed that the expression of ADAT2 was significantly increased in MT BRCA1 tumors compared to WT BRCA1 tumors (Figure 5a,e), consistent with ADAT2 upregulation in BRCA1-depleted mammary cell lines. Conversely, staining scores of CCNL2, DBF4B, and TRIM45 were significantly decreased in MT BRCA1 tumors, supporting the above findings of a downregulation of these proteins in BRCA1-depleted cells (Figure 5b–e). 

Interestingly, ADAT2 appeared as the sole protein to be enriched in BRCA1-deficient tumors and was readily measured by IHC. This raised the possibility that ADAT2 could constitute a candidate biomarker of BRCA1 deficiency, since determining the BRCA1 status remains an issue. We analyzed ADAT2 protein levels in a validation cohort of 13 tumor samples from MT BRCA1 patients (Figure 5a and Table 1) and found that ADAT2 staining was statistically different compared to the control group (WT BRCA1), confirming that ADAT2 high expression was correlated with low expression of BRCA1 in breast tumor samples.

## 4. Discussion

We herein demonstrated that the BRCA1 protein was a translational regulator, which controlled the translational efficiency of a subset of mRNAs with which it associates.

To our knowledge, genome-wide association of mRNA with BRCA1 has not been described previously. In addition, few studies performing small scale analysis of BRCA1-dependent RIP identified some BRCA1-bound RNA [25,26,27]. However, no direct association with BRCA1 has been shown until now as two large scale analyses searching for RNA binding proteins by using UV-crosslink failed to identify BRCA1 among the proteins identified [28,29]. These data and our results underscored the importance of further deciphering at the molecular level the ribonucleoprotein complexes in which BRCA1 protein associates to mRNA. A search for particular RNA motif (Figure S2) within BRCA1-bound mRNAs identified 14 motifs among which only two (motif #2 and motif #7) happened to be recognized by the RBPs SRP14 and KHSRP respectively. Thus, it is tempting to speculate that BRCA1 regulates the translation of subsets of mRNA defined by either motif #2 or motif #7, through its interaction with specific translation initiation complex containing either SRP14 or KHSRP.

The 5′untranslated regions (5′UTRs) and 3′untranslated regions (3′UTRs) of mRNA contain cis-regulatory sequences that allow direct binding with trans-regulators and consequently contribute to modulate the translational efficiency of the corresponding mRNA [30]. Among them, some are secondary structures such as Internal Ribosome Entry Sites, stem-loops, and RNA G-quadruplex. Thus, it will be worth examining the features of the 5′UTR and the 3′UTR of the mRNA translationally controlled by and associated to the BRCA1 protein, to further decipher at the molecular level the mode of action of BRCA1. Especially ribonucleoprotein complexes containing both the BRCA1 protein and CCNL2, DBF4B, TRIM45, or ADAT2 mRNA will need deep scrutiny to shed light on the molecular mechanism through which BRCA1 regulates the translational efficiency of these mRNA.

Low BRCA1 expression in cells along with BRCA1 inactivation in breast tumors is associated with reduced expression of CCNL2, DBF4B, and TRIM45. These proteins are respectively implicated in mRNA splicing and apoptosis, DNA replication, and cell cycle regulation, transcription repression of pro-proliferative signaling, all biological functions known to contribute to the tumor suppressive activity of BRCA1.

Firstly, CCNL2 transcriptionally regulates pre-mRNA splicing [31,32]. Its overexpression induces apoptosis of human hepatocellular and gastric carcinoma cell lines [31,33], while its knockdown promotes growth of pulmonary artery smooth muscle cells [34]. A recent preclinical study conducted in breast cancer cell lines and tissue arrays reported that CCNL2 mRNA transcript expression was lower in breast cancer than in normal breast tissue [35]. Therefore, positively controlling CCNL2 translation may represent a novel mechanism through which BRCA1 exerts its tumor suppressive activity.

Secondly, DBF4B is required for the efficient progression of S and M phases [36], and for DNA replication [37]. Accordingly, DBF4B knockdown leads to an increase in DNA damage in HeLa cells and affects cell cycle in the *Xenopus laevis* model [38]. Therefore, BRCA1 may control DNA replication and S phase progression in response to DNA damage, two hallmarks of its tumor suppressor activity, through at least in part positive regulation of DBF4B translation.

Thirdly, TRIM45 belongs to the family of tripartite motif proteins that play important roles in cell proliferation, differentiation, and apoptosis. TRIM45 suppresses cell proliferation by negatively regulating the mitogen-activated protein kinase signaling pathway [39] and the transcriptional activity of NF-kB [40]. In a large-scale gene expression array analysis conducted in breast cancer samples from Taiwanese women, TRIM45 expression was diminished [41]. Consistently, TRIM45 was recently identified as a tumor suppressor in the brain [42]. Here, the translational control of TRIM45 by BRCA1 that leads to low TRIM45 levels in BRCA1-deficient breast tumors, may represent a novel mechanism contributing to the onco-suppressive role of BRCA1.

Our most intriguing finding was however the repression of ADAT2 expression and function by BRCA1. ADAT2 is the catalytic subunit of the heterodimer ADAT2/ADAT3 responsible for the post-transcriptional modification of tRNA at its first anticodon (wobble) position (position 34). The inosine, (I34), which is generated from adenosine, can translate not only codons ending in uracil, but also those ending in cytosine or adenine. This modification enlarges the codon recognition capacity during protein synthesis [43,44]. We previously suggested that ADAT might be controlling specific genetic programs [45]. Therefore, the BRCA1-driven negative regulation of ADAT2 may silence specific genetic programs the codon usage of which makes their translation dependent on tRNAs with I34 [46]. Emerging evidence indicates that tRNA expression and tRNA repertoire are modulated during tumorigenesis [47,48]. Perturbations of a number of tRNA modifications and altered expression of the corresponding modifying enzymes such as TRMT12, NSUN2, or DNMT2, have been linked to numerous human diseases, including cancer, neurological disorders, and mitochondrial-linked disorders [49,50]. Thus, ADAT2 up-regulation observed upon BRCA1 inactivation in breast tumors exposes A34-to-I34 editing as a novel tRNA modification that is altered in cancer.

These findings raise the possibility of using ADAT2 as a predictive biomarker of efficacy of anti-PARP-based therapy. Indeed, since PARP inhibitors are now available for the treatment of metastatic breast cancer [51], ADAT2 could be used as a predictor of efficacy of such treatments. Of course, this would need further exploration, in particular in patients with BRCA1 pathogenic variant in the tumor (i.e., somatic BRCA1 pathogenic variant).

The anti-PARP-based therapy recently proposed to patients with BRCA1 deficiency makes the identification of BRCA1 pathogenic variant carriers essential. Today, BRCA1 gene sequencing remains the mainstay of BRCA1 pathogenic variants identification [52]. However, widespread BRCA1 genetic testing in unselected individuals is hardly feasible [53]. A simple IHC assay to monitor BRCA1 deficiency directly in tumor biopsies could be more convenient and affordable [54]. So far, none of the many attempts of setting up IHC BRCA1 staining to assign the tumor BRCA1 status has been successful [55,56,57,58]. Our analysis shows a correlation between the high level of ADAT2 and the BRCA1 deficiency among BRCA1 deficient breast tumor samples. Therefore, it could be worth examining further whether detection of ADAT2 by IHC could be proposed as a first screen for BRCA1 deficiency. Moreover, sporadic breast cancer cases, which harbor BRCA1 deficiency, may benefit from this IHC since they become more and more important in clinical practice, with respect to anti-PARP based therapy. Nevertheless, our analysis is based on a relatively small sample size and so further validation on a large cohort will be required to fully assess the potential of ADAT2 as a marker of BRCA1 deficiency.

BRCA1-mediated ADAT2 regulation may contribute to increase the diversity of the proteome. In addition we have shown that BRCA1 regulates protein synthesis [17] and it was reported that by its interaction with RNA Polymerase I and RNA Polymerase III machineries, BRCA1 could regulate synthesis of rRNA and tRNA [59,60]. This strongly supports the notion that BRCA1 is a translational regulator, a key function for its tumor suppressive activity as it has been shown for the P53 tumor suppressor [61,62].

## 5. Conclusions

In this study, we provided evidence that BRCA1 altered translational efficiency of subsets of mRNAs involved in major programs in human tumors. The BRCA1 protein associated with these mRNA in ribonucleoprotein complexes and modulated their translational efficiency. We focused on ADAT2, CCNL2, DBF4B, and TRIM45 mRNAs that played key roles in cancer biology to demonstrate that alteration of their translational efficiency occurred via a BRCA1-dependent mechanism in cultured cells and in patient tumors. This study raised the possibility of using ADAT2 as a predictive biomarker of efficacy of anti-PARP-based therapy. These findings are crucial not only for basic research but also for clinical outcome since most BRCA1 mutated tumors are defined as “triple negative breast tumors”, which are associated with a very poor prognosis.

## Figures and Tables

**Table 1:** Clinical and tumor characteristics of breast carcinoma tissues.

	Exploratory Cohort		Validation Cohort
Characteristics	WT BRCA1	MT BRCA1	MT BRCA1
	*n* = 26	*n* = 19	*n* = 13
**Tumor Type (%)**			
Invasive ductal carcinoma	26 (100)	19 (100)	13 (100)
**SBR Grade (%)**			
1	0 (0)	0 (0)	0 (0)
2	8 (31)	0 (0)	2 (15)
3	18 (69)	19 (100)	11 (85)
**Subtype (%)**			
HR negative HER2 negative	17 (65)	13 (68)	10 (77)
HR positive HER2 negative	9 (35)	6 (32)	3 (23)
**Age (year)**			
Median (min-max)	43 (28–65)	38 (23–61)	43 (36–65)

## Supplementary Materials

The following are available online at https://www.mdpi.com/2073-4409/9/4/941/s1, Supplementary Material and Methods, Figure S1: RT-qPCR analyses of differentially translated mRNA upon BRCA1 depletion, Figure S2: Search for RNA motifs present in the 488 RNA interacting with BRCA1, Table S1: Primers used for qPCR.

## References

[1] Bray F., Ferlay J., Soerjomataram I., Siegel R.L., Torre L.A., Jemal A. (2018). Global cancer statistics 2018: GLOBOCAN estimates of incidence and mortality worldwide for 36 cancers in 185 countries. Ca A Cancer J. Clin..

[2] Malone K.E., Daling J.R., Doody D.R., Hsu L., Bernstein L., Coates R.J., Marchbanks P.A., Simon M.S., McDonald J.A., Norman S.A. (2006). Prevalence and predictors of BRCA1 and BRCA2 mutations in a population-based study of breast cancer in white and black American women ages 35 to 64 years. Cancer Res..

[3] Kurian A.W., Gong G.D., John E.M., Miron A., Felberg A., Phipps A.I., West D.W., Whittemore A.S. (2009). Performance of prediction models for BRCA mutation carriage in three racial/ethnic groups: Findings from the Northern California Breast Cancer Family Registry. Cancer Epidemiol. Biomark. Prev..

[4] Armstrong N., Ryder S., Forbes C., Ross J., Quek R.G. (2019). A systematic review of the international prevalence of BRCA mutation in breast cancer. Clin. Epidemiol..

[5] Kuchenbaecker K.B., Hopper J.L., Barnes D.R., Phillips K.A., Mooij T.M., Roos-Blom M.J., Jervis S., van Leeuwen F.E., Milne R.L., Andrieu N. (2017). Risks of Breast, Ovarian, and Contralateral Breast Cancer for BRCA1 and BRCA2 Mutation Carriers. JAMA.

[6] Engel C., Fischer C., Zachariae S., Bucksch K., Rhiem K., Giesecke J., Herold N., Wappenschmidt B., Hubbel V., Maringa M. (2019). Breast cancer risk in BRCA1/2 mutation carriers and noncarriers under prospective intensified surveillance. Int. J. Cancer.

[7] Nik-Zainal S., Davies H., Staaf J., Ramakrishna M., Glodzik D., Zou X., Martincorena I., Alexandrov L.B., Martin S., Wedge D.C. (2016). Landscape of somatic mutations in 560 breast cancer whole-genome sequences. Nature.

[8] Staff S., Isola J., Tanner M. (2003). Haplo-insufficiency of BRCA1 in sporadic breast cancer. Cancer Res..

[9] Atchley D.P., Albarracin C.T., Lopez A., Valero V., Amos C.I., Gonzalez-Angulo A.M., Hortobagyi G.N., Arun B.K. (2008). Clinical and pathologic characteristics of patients with BRCA-positive and BRCA-negative breast cancer. J. Clin. Oncol..

[10] Robson M., Im S.A., Senkus E., Xu B., Domchek S.M., Masuda N., Delaloge S., Li W., Tung N., Armstrong A. (2017). Olaparib for Metastatic Breast Cancer in Patients with a Germline BRCA Mutation. N. Engl. J. Med..

[11] Venkitaraman A.R. (2014). Cancer suppression by the chromosome custodians, BRCA1 and BRCA2. Science.

[12] Silver D.P., Livingston D.M. (2012). Mechanisms of BRCA1 tumor suppression. Cancer Discov..

[13] Roy R., Chun J., Powell S.N. (2011). BRCA1 and BRCA2: Different roles in a common pathway of genome protection. Nat. Rev. Cancer.

[14] Pathania S., Bade S., Le Guillou M., Burke K., Reed R., Bowman-Colin C., Su Y., Ting D.T., Polyak K., Richardson A.L. (2014). BRCA1 haploinsufficiency for replication stress suppression in primary cells. Nat. Commun..

[15] Zhang X., Chiang H.C., Wang Y., Zhang C., Smith S., Zhao X., Nair S.J., Michalek J., Jatoi I., Lautner M. (2017). Attenuation of RNA polymerase II pausing mitigates BRCA1-associated R-loop accumulation and tumorigenesis. Nat. Commun..

[16] Savage K.I., Harkin D.P. (2015). BRCA1, a ‘complex’ protein involved in the maintenance of genomic stability. Febs J..

[17] Dizin E., Gressier C., Magnard C., Ray H., Decimo D., Ohlmann T., Dalla Venezia N. (2006). BRCA1 interacts with poly(A)-binding protein: Implication of BRCA1 in translation regulation. J. Biol. Chem..

[18] Dacheux E., Vincent A., Nazaret N., Combet C., Wierinckx A., Mazoyer S., Diaz J.J., Lachuer J., Venezia N.D. (2013). BRCA1-Dependent Translational Regulation in Breast Cancer Cells. PLoS ONE.

[19] Eisinger F., Bressac B., Castaigne D., Cottu P.H., Lansac J., Lefranc J.P., Lesur A., Nogues C., Pierret J., Puy-Pernias S. (2004). Identification and management of hereditary predisposition to cancer of the breast and the ovary (update 2004). Bull. Du Cancer.

[20] Vincent A., Berthel E., Dacheux E., Magnard C., Venezia N.L. (2016). BRCA1 affects protein phosphatase 6 signalling through its interaction with ANKRD28. Biochem. J..

[21] Keene J.D., Komisarow J.M., Friedersdorf M.B. (2006). RIP-Chip: The isolation and identification of mRNAs, microRNAs and protein components of ribonucleoprotein complexes from cell extracts. Nat. Protoc..

[22] Huang da W., Sherman B.T., Lempicki R.A. (2009). Systematic and integrative analysis of large gene lists using DAVID bioinformatics resources. Nat. Protoc..

[23] Torres A.G., Pineyro D., Rodriguez-Escriba M., Camacho N., Reina O., Saint-Leger A., Filonava L., Batlle E., Ribas de Pouplana L. (2015). Inosine modifications in human tRNAs are incorporated at the precursor tRNA level. Nucleic Acids Res..

[24] Wulff T.F., Arguello R.J., Molina Jordan M., Roura Frigole H., Hauquier G., Filonava L., Camacho N., Gatti E., Pierre P., Ribas de Pouplana L. (2017). Detection of a Subset of Posttranscriptional Transfer RNA Modifications in Vivo with a Restriction Fragment Length Polymorphism-Based Method. Biochemistry.

[25] Kawai S., Amano A. (2012). BRCA1 regulates microRNA biogenesis via the DROSHA microprocessor complex. J. Cell Biol..

[26] Savage K.I., Gorski J.J., Barros E.M., Irwin G.W., Manti L., Powell A.J., Pellagatti A., Lukashchuk N., McCance D.J., McCluggage W.G. (2014). Identification of a BRCA1-mRNA splicing complex required for efficient DNA repair and maintenance of genomic stability. Mol. Cell.

[27] Sharma V., Khurana S., Kubben N., Abdelmohsen K., Oberdoerffer P., Gorospe M., Misteli T. (2015). A BRCA1-interacting lncRNA regulates homologous recombination. Embo Rep..

[28] Baltz A.G., Munschauer M., Schwanhausser B., Vasile A., Murakawa Y., Schueler M., Youngs N., Penfold-Brown D., Drew K., Milek M. (2012). The mRNA-bound proteome and its global occupancy profile on protein-coding transcripts. Mol. Cell.

[29] Castello A., Fischer B., Eichelbaum K., Horos R., Beckmann B.M., Strein C., Davey N.E., Humphreys D.T., Preiss T., Steinmetz L.M. (2012). Insights into RNA biology from an atlas of mammalian mRNA-binding proteins. Cell.

[30] Jackson R.J., Hellen C.U., Pestova T.V. (2010). The mechanism of eukaryotic translation initiation and principles of its regulation. Nat. Rev. Mol. Cell Biol..

[31] Yang L., Li N., Wang C., Yu Y., Yuan L., Zhang M., Cao X. (2004). Cyclin L2, a novel RNA polymerase II-associated cyclin, is involved in pre-mRNA splicing and induces apoptosis of human hepatocellular carcinoma cells. J. Biol. Chem..

[32] Loyer P., Trembley J.H., Grenet J.A., Busson A., Corlu A., Zhao W., Kocak M., Kidd V.J., Lahti J.M. (2008). Characterization of cyclin L1 and L2 interactions with CDK11 and splicing factors: Influence of cyclin L isoforms on splice site selection. J. Biol. Chem..

[33] Li H.L., Huang D.Z., Deng T., Zhou L.K., Wang X., Bai M., Ba Y. (2012). Overexpression of cyclin L2 inhibits growth and enhances chemosensitivity in human gastric cancer cells. Asian Pac. J. Cancer Prev..

[34] Liu H., Tao Y., Chen M., Yu J., Li W.J., Tao L., Li Y., Li F. (2016). Upregulation of MicroRNA-214 Contributes to the Development of Vascular Remodeling in Hypoxia-induced Pulmonary Hypertension Via Targeting CCNL2. Sci. Rep..

[35] Kren B.T., Unger G.M., Abedin M.J., Vogel R.I., Henzler C.M., Ahmed K., Trembley J.H. (2015). Preclinical evaluation of cyclin dependent kinase 11 and casein kinase 2 survival kinases as RNA interference targets for triple negative breast cancer therapy. Breast Cancer Res..

[36] Yoshizawa-Sugata N., Ishii A., Taniyama C., Matsui E., Arai K., Masai H. (2005). A second human Dbf4/ASK-related protein, Drf1/ASKL1, is required for efficient progression of S and M phases. J. Biol. Chem..

[37] Takahashi T.S., Walter J.C. (2005). Cdc7-Drf1 is a developmentally regulated protein kinase required for the initiation of vertebrate DNA replication. Genes Dev..

[38] Collart C., Smith J.C., Zegerman P. (2017). Chk1 Inhibition of the Replication Factor Drf1 Guarantees Cell-Cycle Elongation at the Xenopus laevis Mid-blastula Transition. Dev. Cell.

[39] Wang Y., Li Y., Qi X., Yuan W., Ai J., Zhu C., Cao L., Yang H., Liu F., Wu X. (2004). TRIM45, a novel human RBCC/TRIM protein, inhibits transcriptional activities of ElK-1 and AP-1. Biochem. Biophys. Res. Commun..

[40] Shibata M., Sato T., Nukiwa R., Ariga T., Hatakeyama S. (2012). TRIM45 negatively regulates NF-kappaB-mediated transcription and suppresses cell proliferation. Biochem. Biophys. Res. Commun..

[41] Huang C.C., Tu S.H., Lien H.H., Jeng J.Y., Huang C.S., Huang C.J., Lai L.C., Chuang E.Y. (2013). Concurrent gene signatures for han chinese breast cancers. PLoS ONE.

[42] Zhang J., Zhang C., Cui J., Ou J., Han J., Qin Y., Zhi F., Wang R.F. (2017). TRIM45 functions as a tumor suppressor in the brain via its E3 ligase activity by stabilizing p53 through K63-linked ubiquitination. Cell Death Dis..

[43] Gerber A.P., Keller W. (1999). An adenosine deaminase that generates inosine at the wobble position of tRNAs. Science.

[44] Novoa E.M., Pavon-Eternod M., Pan T., Ribas de Pouplana L. (2012). A role for tRNA modifications in genome structure and codon usage. Cell.

[45] Torres A.G., Pineyro D., Filonava L., Stracker T.H., Batlle E., Ribas de Pouplana L. (2014). A-to-I editing on tRNAs: Biochemical, biological and evolutionary implications. Febs Lett..

[46] Rafels-Ybern A., Torres A.G., Grau-Bove X., Ruiz-Trillo I., Ribas de Pouplana L. (2017). Codon adaptation to tRNAs with Inosine modification at position 34 is widespread among Eukaryotes and present in two Bacterial phyla. Rna Biol..

[47] Pavon-Eternod M., Gomes S., Geslain R., Dai Q., Rosner M.R., Pan T. (2009). tRNA over-expression in breast cancer and functional consequences. Nucleic Acids Res..

[48] Goodarzi H., Nguyen H.C.B., Zhang S., Dill B.D., Molina H., Tavazoie S.F. (2016). Modulated Expression of Specific tRNAs Drives Gene Expression and Cancer Progression. Cell.

[49] Kirchner S., Ignatova Z. (2015). Emerging roles of tRNA in adaptive translation, signalling dynamics and disease. Nat. Rev. Genet..

[50] Torres A.G., Batlle E., Ribas de Pouplana L. (2014). Role of tRNA modifications in human diseases. Trends Mol. Med..

[51] Litton J.K., Rugo H.S., Ettl J., Hurvitz S.A., Goncalves A., Lee K.H., Fehrenbacher L., Yerushalmi R., Mina L.A., Martin M. (2018). Talazoparib in Patients with Advanced Breast Cancer and a Germline BRCA Mutation. N. Engl. J. Med..

[52] Popova T., Manie E., Rieunier G., Caux-Moncoutier V., Tirapo C., Dubois T., Delattre O., Sigal-Zafrani B., Bollet M., Longy M. (2012). Ploidy and large-scale genomic instability consistently identify basal-like breast carcinomas with BRCA1/2 inactivation. Cancer Res..

[53] Katz S.J., Ward K.C., Hamilton A.S., McLeod M.C., Wallner L.P., Morrow M., Jagsi R., Hawley S.T., Kurian A.W. (2018). Gaps in Receipt of Clinically Indicated Genetic Counseling After Diagnosis of Breast Cancer. J. Clin. Oncol..

[54] Bankhead P., Fernandez J.A., McArt D.G., Boyle D.P., Li G., Loughrey M.B., Irwin G.W., Harkin D.P., James J.A., McQuaid S. (2018). Integrated tumor identification and automated scoring minimizes pathologist involvement and provides new insights to key biomarkers in breast cancer. Lab. Invest..

[55] Vorrius T.R., Snyder K., Pica-Mendez A., Tan C., Laterza O., Toniatti C., Carpenter C., Lee H., Tanaka W., Zhang Z.Q. (2009). Immunohistochemical Detection of BRCA-1 and BRCA-2 Expression in Human Breast and Ovarian Tumors. J. Histotechnol..

[56] Perez-Valles A., Martorell-Cebollada M., Nogueira-Vazquez E., Garcia-Garcia J.A., Fuster-Diana E. (2001). The usefulness of antibodies to the BRCA1 protein in detecting the mutated BRCA1 gene. An immunohistochemical study. J. Clin. Pathol..

[57] Milner R., Wombwell H., Eckersley S., Barnes D., Warwicker J., Van Dorp E., Rowlinson R., Dearden S., Hughes G., Harbron C. (2013). Validation of the BRCA1 antibody MS110 and the utility of BRCA1 as a patient selection biomarker in immunohistochemical analysis of breast and ovarian tumours. Virchows Arch..

[58] Mangia A., Chiriatti A., Tommasi S., Menolascina F., Petroni S., Zito F.A., Simone G., Schittulli F., Paradiso A. (2009). BRCA1 expression and molecular alterations in familial breast cancer. Histol. Histopathol..

[59] Johnston R., D’Costa Z., Ray S., Gorski J., Harkin D.P., Mullan P., Panov K.I. (2016). The identification of a novel role for BRCA1 in regulating RNA polymerase I transcription. Oncotarget.

[60] Veras I., Rosen E.M., Schramm L. (2009). Inhibition of RNA polymerase III transcription by BRCA1. J. Mol. Biol..

[61] Marcel V., Catez F., Diaz J.J. (2015). p53, a translational regulator: Contribution to its tumour-suppressor activity. Oncogene.

[62] Marcel V., Nguyen Van Long F., Diaz J.J. (2018). 40 Years of Research Put p53 in Translation. Cancers.

