# Supplementary material for: Uncovering the Translational Regulatory Activity of the Tumor Suppressor BRCA1
Source: Cells. 2020 Apr 10;9(4):941. doi: 10.3390/cells9040941 (PMC7226996; doi:10.3390/cells9040941)
Supplement: Supplementary file 1 [file cells-09-00941-s001.pdf]

Supplemental Material and Methods

Chemicals

The primary antibodies used for immunoblotting were as follows: mouse monoclonal antibodies to BRCA1 (MS110, Calbiochem), β-Actin (AC-15, Sigma) and α-Tubulin (DM1A, Calbiochem), rabbit polyclonal antibodies for ADAT2 (ab135429, Abcam), CCNL2 (A301-677A, Bethyl), DBF4B (ab177911, Abcam), TRIM45 (ab84303, Abcam). Secondary antibodies used were peroxydase-conjugated anti-mouse or anti-rabbit immunoglobulins (Jackson ImmunoResearch).

Case selection

Among these patients with such a medical history, we set up a cohort of patients with documented germ-line BRCA1 and BRCA2 pathogenic variant statuses, as determined by Next Generation Sequencing (NGS) in blood samples. Variants were classified as pathogenic according to the ENIGMA BRCA1/2 Gene Variant Classification Criteria (http://www.enigmaconsortium.org/). Women with variants of uncertain significance were considered as non-carriers. All patients were treated at the Regional Cancer Hospital "Centre Léon Bérard" (CLB) for breast adenocarcinoma after 2006. All patients gave written consent for the use of their tumor tissue. All patients that are undergoing tissue sampling (including surgery) in our institution have to give their consent for the use of the samples for research purposes (Institutional Ethics Committee). All patient data were completely de-identified before access and analysis. This was a monocentric study and the institution provided consent to use biological samples that were collected as part of routine patient care. Under French law, the study is therefore exempt from prior authorization by the CNIL. This study has been approved by the Institutional Ethics Committee from the Centre Léon Bérard.

Patients were only included in our cohort if they had not received any neo-adjuvant treatment before breast surgery. Patients carrying a BRCA2 pathogenic variant were excluded (i.e. all patients in our cohort were BRCA2 wild-type). Patients for whom tumor samples were not suitable for TMA analysis were excluded as well.

We first set up an exploratory cohort including two groups of patients: patients with a BRCA1 pathogenic variant constituted the "MT BRCA1" group (n = 19), while those without made up the "WT BRCA1" group (n = 26). Whenever possible, both groups were matched according to their histopathological tumor SBR grade (Scarff Bloom and Richardson), hormone receptor (HR) status, and HER2 status. As expected, patients with BRCA1 pathogenic variant where younger and had a higher frequency of triple negative tumors, compared with WT BRCA1. The aim of this exploratory cohort was to detect "a tendency" between the two groups. ADAT2 was subsequently identified as the best antibody for further exploration (please, refer to "Results" section). To confirm the potential value of this antibody, we set up a validation cohort of 13 additional independent patients, selected following the same methodology as described above.

Tissue microarray (TMA) and immunohistochemistry (IHC)

The slides were incubated on a BenchMark ULTRA automate (Roche Diagnostics, Meylan, France) for deparaffinization and inhibition of endogenous peroxidases in H2O2 using the ULTRA view universal DAB kit (Roche, ref:760-500, Meylan, France). The fully automated workflow of the BenchMark ULTRA automate enabled processing of a maximum of 30 slides simultaneously. The IHC staining was therefore performed using batch of antibodies necessary for labelling 30 slides at most.

Heat-induced antigen retrieval was performed in CC1 buffer (pH 8.4) (950-224, Roche) for 64 min on the BenchMark ULTRA automate for rabbit polyclonal antibodies against ADAT2 (ab122280, Abcam), DBF4B (NBP2-13900, Novus Biologicals) and TRIM45 (NBP1-82266, Novus Biologicals). Rabbit polyclonal antibody against CCNL2 (NBP2-47497, Novus Biologicals) did not require antigen retrieval. Anti-ADAT2 was diluted at 1/100, anti-CCNL2 at 1/25, anti-DBF4B at 1/50 and anti-TRIM45 at 1/100.

The slides were incubated with the primary antibody on the BenchMark ULTRA automate for 60 min at 37°C, except for the anti-DBF4B antibody which was incubated for 60 min at room temperature.
Antibody binding was detected using the Ultra view Universal DAB Detection kit (760-500, Roche) for all antibodies: secondary antibodies were incubated for 8 min at 37°C followed by the DAB substrate for another 8 min incubation at 37°C. Regarding the anti-CCNL2 and anti-TRIM45 antibodies, an additional step using the Amplification kit (760-080, Roche) was performed before antibody binding detection using the protocol provided by the supplier.
Sections were counterstained with Hematoxylin (760-2021, Roche), and bluing (760-2037, Roche). Then, the slides were analyzed by a senior pathologist experienced in breast pathology who assessed in the invasive carcinoma, the percentage and intensity of cytoplasmic staining for anti-ADAT2, anti-DBF4B, anti-TRIM45 and the percentage and intensity of both cytoplasmic and nuclear staining for anti-CCNL2.

Cell culture
The human breast carcinoma MDA-MB-231 (basal, ER-, PR-) and MCF-7 (luminal, ER+, PR+) cell lines (available from the ATCC) were maintained in Dulbecco's Modified Eagle's Medium (DMEM) containing 4.5 g/l of glucose supplemented with 10 % fetal calf serum, 100 µg/ml streptomycin and 100 units/ml of penicillin. For MCF-7 cell line, 0.1 mM non-essential amino acids, 1 mM sodium-pyruvate and 0.01 mg/ml recombinant human insulin were added to the medium.
Normal human mammary epithelial cells (HMECs) (available from the ATCC) were maintained in DMEM-F12 supplemented with 0.5 µg/ml hydrocortisone, 10 ng/ml EGF, 0.3 U/ml insulin, 10% fetal calf serum, 100 µg/ml streptomycin and 100 units/ml of penicillin.

Immunoblotting
The MCF-7, MDA-MB-231 or HMEC cells were lysed in lysis buffer A (20 mM Hepes-KOH pH 7.2, 100 mM KCl, 1 mM DTT, 0.5 mM EDTA, 0.5 % NP40, 10 % Glycerol) supplemented with protease inhibitor (Complete EDTA free, Roche). Proteins were subjected to SDS-PAGE, and blotted onto poly-vinylidene difluoride (PVDF) membranes (Immobilon-P, Millipore). Membranes were blocked in Tris-buffered saline solution containing 0.05 % Tween 20 and 5 % non-fat milk and incubated with primary antibodies. Horseradish peroxidase conjugated secondary antibodies (Jackson ImmunoResearch) were used for the detection of immunoreactive proteins by chemiluminescence (ECL, GE Healthcare). We performed this experiment first in MCF-7 cells with which we identified mRNA that were specifically deregulated under BRCA1 deficiency (please, refer to "Results" section). Then, we enlarged our investigation to two additional cell lines: (i) the MDA-MB-231 mammary cell line because it presents opposite characteristics to MCF-7 cell line; (ii) the HMEC cell line because it is representative of normal human mammary epithelial cells.

Isolation of polysomes and total cytoplasmic RNA
Extracts from MCF-7 cells were prepared by lysis at 4°C in extraction buffer (50 mM Tris-HCl, pH 7.4, 25 mM KCl, 5 mM MgCl2, 250 mM sucrose, 0.7% Nonidet-P40, and 100 µg/ml cycloheximide). After elimination of nuclei and mitochondria by centrifugation, the supernatant (cytoplasmic fraction) was divided in two. One fifth was used to extract total cytoplasmic RNA and the remaining 80 % were layered onto a linear 10-40 % sucrose gradient. A 2 h centrifugation at 250 000 g and at 4°C in a SW41Ti rotor (Beckman) was then performed. The gradients were collected in 14 fractions and absorbance profiles were generated at 254 nm using an ISCO UA-6 detector. Polysomal fractions (fractions 7 to 14) were pooled for further RNA extraction using Trizol LS Reagent as described by the manufacturer (Life technologies).

RNA-binding protein immunoprecipitation (RIP)
The MCF-7 cells were washed with ice-cold PBS and lysed in RIP buffer (25 mM Tris-HCl at pH 7.4, 150 mM KCl, 2 mM EDTA, 0.5 % NP40, 0.5 mM DTT, 200 U/ml RNaseOut, protease and phosphatase inhibitors). Where indicated, a small fraction of the lysate (5 %) was used to prepare total RNA extracts

and this was considered the input. The remaining lysates were then split into equal parts: half was incubated overnight at 4°C with mouse monoclonal anti-BRCA1 antibody (BRCA1) (MS110, Calbiochem) coupled to Dynabeads (Life Technologies) and half was incubated with ChIP Grade mouse control IgG (NR) (ab18413, Abcam) used as control and coupled to Dynabeads. Immunoprecipitates were washed seven times with RIPA buffer (50 mM Tris-HCl at pH 7.4, 150 mM KCl, 0.5 % NP40). The RNA associated with each type of beads, and the total RNA (input) were then eluted with elution buffer (50 mM Tris-HCl at pH 7.4, 5 mM EDTA, 10 mM DTT, 1 % SDS, 200 U/ml RNase Out) and digested with proteinase K. RNA was finally extracted using the RNeasy micro kit (Qiagen).

Microarray analysis
Microarray analysis was performed using a high-density oligonucleotide array (Human Transcriptome Array GeneChip HG-ST 2.0, Affymetrix, Santa Clara, CA, USA). RNA (900 pg) was reverse-transcribed and pre-amplified with the Ovation WTA System (Nugen). cDNA quantification was performed with a nanodrop and quality checked with the Agilent 2100 Bioanalyzer (Agilent technologies, Inc, Palto Alto, CA, USA). Fragmentation and labeling of cDNA (3.6 μg) using Encore Biotin Module kit (Nugen) were performed. Before amplification, spikes of synthetic mRNA at different concentrations were added to all samples; these positive controls were used to ascertain the quality of the process. After final purification, hybridization was performed following Affymetrix protocols (http://www.affymetrix.com). The arrays were read with a confocal laser (GeneChip Scanner 3000 7G, Affymetrix). Then CEL files were generated using the Affymetrix GeneChip Command Console (AGCC) software 3.0.

Quantification of A-to-I editing
A 50 nucleotide single-end sequencing was done. Analyses were performed in duplicate. 4 samples (2 Control and 2 BRCA1-overexpression samples) were run in one single sequencing lane obtaining approximately 50-70 million reads per sample. However, we were only able to obtain sufficient reads mapping to the anticodon of tRNA substrates of ADAT for tRNASerAGA and tRNAValAAC (at least 100 reads having I34 in the Control samples); therefore, A-to-I editing was exclusively evaluated for those two substrates.

Statistical analysis
Statistical analyses were performed using GraphPad Prism 5.0a (GraphPad Software, Inc). Correlation between fold enrichment obtained by RT-qPCR (Fe) and the fold change obtained by microarray (Fc) or total RNA levels (Inputs) were tested using the non-parametric Spearman r test. For the A-to-I editing experiments, we used a Fisher's Exact test with Benjamini-Hochberg adjusted p-values as previously described [23]. For IHC analyses, enrichment in low and high staining scores was investigated using the Chi-square test. P-values < 0.05 were considered statistically significant. Asterisks denote statistical significance (* $p < 0.05$; ** $p < 0.01$; *** $p < 0.001$).
Standard errors of the means (SEM) shown in figures are the standard deviations for the samples divided by the square root of the sample size. For results shown in each figure, except figures 4f and 4g, at least three independent experiments were performed, ensuring statistical independence of the values in the sample.

a

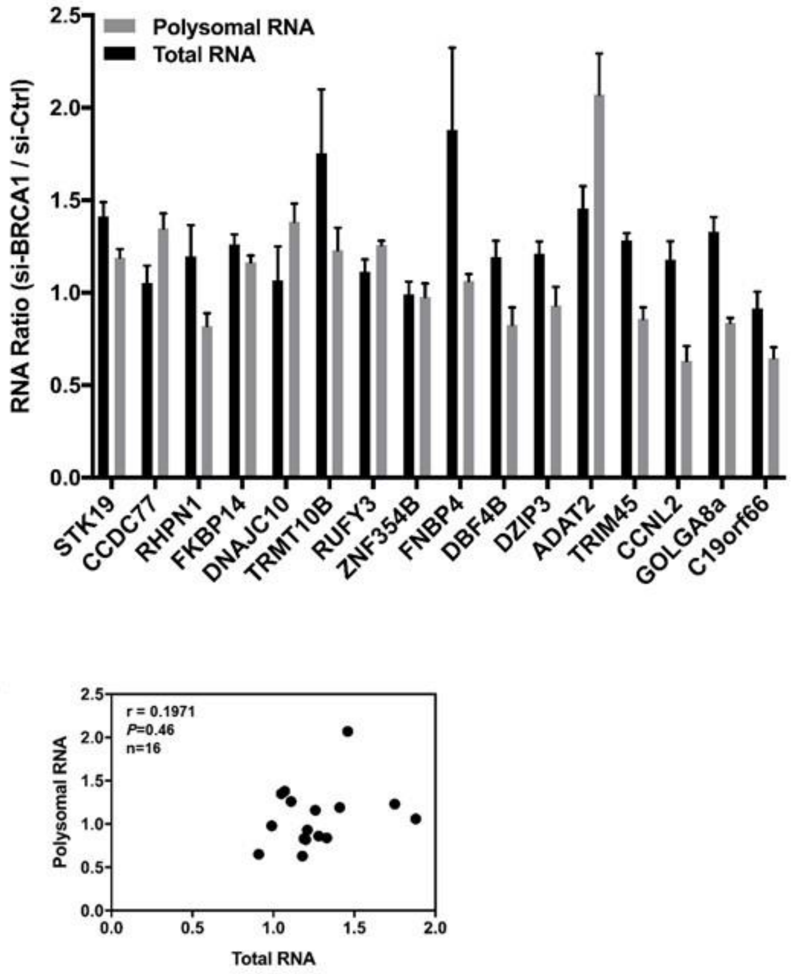

Figure S1: RT-qPCR analyses of differentially translated mRNA upon BRCA1 depletion.
a For each gene, the ratios for polysomal RNA (polyRNA = si-BRCA1/si-Ctrl) (grey) and for total RNA (totRNA = si-BRCA1/si-Ctrl) (black) were calculated. Data are expressed as means ± SEM. N = 3. **b** Comparison of polysomal and total RNA levels. Mean ratios for polysomal RNA and mean ratios for total RNA were plotted and their correlation was assessed using the Spearman test. No significant correlation was observed.

a

Motif ID	Motif Sequence	Number of mRNA	E-value
1	CGCSCGGC	404	1.8e-098
2	CGCCTGTA	400	1.6e-086
3	CCGCCKCG	381	4.6e-077
4	ACCCCGTC	394	3.8e-071
5	CGCGCCRC	333	5.7e-071
6	CGAGTAGC	364	4.6e-070
7	CCGCCKCC	464	7.9e-066
8	CGAGACCA	387	1.3e-060
9	CACCRCGC	419	2.4e-059
10	ATCTCGGC	374	2.3e-058
11	CGCCCGCS	339	7.9e-053
12	TAAAAATA	440	1.6e-052
13	GAGACGGA	362	7.2e-052
14	AGAATCGC	347	3.4e-051

Legend:
K "Keto" = GT , R "Purine" = AG , S "Strong" = CG

b

Motif	RBP
#2 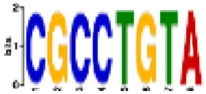	SRP14<br>Signal Recognition Particle 14
#7 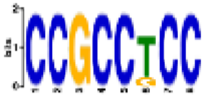	KHSRP<br>KH type-Splicing Regulatory Protein

Figure S2: Search for RNA motifs present in the 488 RNA interacting with BRCA1.
a From Gene Symbol of the 488 genes, the Gene ID (ENSG) were retrieved in Ensembl database, using ensembldb R package (Rainer, J et al, 2019, Bioinformatics) and their sequences were downloaded via Ensembl REST API. Using DREME, 14 motifs were identified to be common to at least 333 genes, with statistically sound values (Bailey, TL, 2011, Bioinformatics).
b Using the ATtRACT database (https://attract.cnic.es/searchmotif) to determine whether one or several of these motifs were known to interact with some protein, two motifs were associated with known RNA Binding Proteins (RBP).

Gene	Primer F (5' > 3')	Primer R (5' > 3')
ACTIN	CTCTTCCAGCCTTCCTTCCT	TGTTGGCGTACAGGTCTTTG
ADAT2	TGTCGTCAAAGTGGCAAGAG	GATTTTCATCAGGCGGAGAG
C19orf66	GGCTCTGATGAGGAAATTCG	TGGGCATCCAGATCGTTACT
CCDC77	AAAAGTGTTGCCCGTTATGC	GGAAATGCACTGCTCTTGGT
CCNL2	TGCCTGCATTTATCTTGCTG	AAGAAACCAATGGGGACGA
DBF4B	GACTCGGTGCCTCTAAGCAG	ACGTGCAGAATCCTCACTCC
DNAJC10	TGCAATGCAGCATGTTAGAAG	CAATACCAGCAGCAAAAGCA
DZIP3	TGCGGTAGACAGTTGGAATG	TGGAGGCAAGGTACTCAAGG
FKBP14	TCGCTGCTTTTGTAGGGACT	GCCCCAATCAAAGAAGTGAC
FNBP4	GGAAGCAGCAACATCTACCC	CCGTGTTCTCATCCCAGACT
GOLGA8a	GATACGCATCGGGTAGAGGA	CACCTCAGAGGACACTGCTG
POP4	GCCAAGCTCTTAAAGGCAGA	CTTTCAGGCGGTCTTCTTTG
RHPN1	GCCCAGGAATGTGTGTTTG	CCGGTGCACTAGCCTGTACT
RUFY3	GGTGATGGAGCACTGTCTGA	GCTGTTATCTCTGCGGCTTC
STK19	CTTGGCCTCACCTACCATGT	GCTGAGCAATGATGAGCAGA
TRIM45	GTTGCCGTTGTCCCTAAAGA	TTGATGCAGACCCACACAGT
TRMT10B	TGTGTGAGGATGAATGATGGA	GGTACACAAGGGTTTCCAAGG
ZNF354B	TGGGAGGTGGAGAAAGACAG	TGAAGTTCCAGGGTTCATCC

Table S1. Primers used for qPCR.
